# Hen uterine gene expression profiling during eggshell formation reveals putative proteins involved in the supply of minerals or in the shell mineralization process

**DOI:** 10.1186/1471-2164-15-220

**Published:** 2014-03-21

**Authors:** Aurélien Brionne, Yves Nys, Christelle Hennequet-Antier, Joël Gautron

**Affiliations:** 1 INRA, UR83 Recherches Avicoles, F-37380 Nouzilly, France

**Keywords:** Chicken, Eggshell, Uterus, Transcriptome, Biomineralization, Mineral supply, Organic matrix

## Abstract

**Background:**

The chicken eggshell is a natural mechanical barrier to protect egg components from physical damage and microbial penetration. Its integrity and strength is critical for the development of the embryo or to ensure for consumers a table egg free of pathogens. This study compared global gene expression in laying hen uterus in the presence or absence of shell calcification in order to characterize gene products involved in the supply of minerals and / or the shell biomineralization process.

**Results:**

Microarrays were used to identify a repertoire of 302 over-expressed genes during shell calcification. GO terms enrichment was performed to provide a global interpretation of the functions of the over-expressed genes, and revealed that the most over-represented proteins are related to reproductive functions. Our analysis identified 16 gene products encoding proteins involved in mineral supply, and allowed updating of the general model describing uterine ion transporters during eggshell calcification. A list of 57 proteins potentially secreted into the uterine fluid to be active in the mineralization process was also established. They were classified according to their potential functions (biomineralization, proteoglycans, molecular chaperone, antimicrobials and proteases/antiproteases).

**Conclusions:**

Our study provides detailed descriptions of genes and corresponding proteins over-expressed when the shell is mineralizing. Some of these proteins involved in the supply of minerals and influencing the shell fabric to protect the egg contents are potentially useful biological markers for the genetic improvement of eggshell quality.

## Background

The chicken egg is a giant reproductive cell constituted of an oocyte surrounded by nutritional reserves; unfertilized eggs contain highly nutritious animal protein which is consumed worldwide. When an egg is fertilized, the eggshell ensures the mechanical protection of the embryo in this closed, self-sufficient and sterile chamber. The laying hen deposits in the egg all components that are essential for the development of a reproductive cell into a mature chick (nutrients, bioactive molecules, protective systems) [1,2]. The egg consists of yolk, white, shell membranes and calcified shell. In order to face physical and microbial assaults, the egg possesses two major defensive mechanisms, a chemical protection system composed of proteins with antimicrobial properties distributed in all compartments [2,3], and the eggshell, which acts as a physical barrier as long as it remains intact [4].

The bird eggshell itself is a complex bioceramic material formed in the uterine (shell gland) segment of the chicken oviduct. It consists of inner and outer eggshell membranes, a calcified zone composed of mammillary layer and a compact palisade layer, covered by a thin outer organic cuticle layer. The shell is a complex biomaterial made of 95% calcium carbonate in its calcitic form and 3.5% organic matrix component, which is a complex mixture of proteins, glycoproteins and proteoglycans [5-9]. The organic and mineral precursors required for eggshell mineralization are secreted daily by the uterus during a period of about 20 hours into a cell-free medium (uterine fluid), which bathes the egg during the 3 phases of shell mineralization (initiation, growth and arrest). This fluid contains all the elements (mineral and organic) necessary for shell formation [10,11]. In the uterine fluid, the organic matrix interacts with minerals and is believed to play a key role in establishing the texture of the shell and its resulting mechanical properties, as observed in other biominerals [12,13]. In hens, this role was confirmed by experimental evidences using *in vitro, in situ* and genomic approaches [4,6,8]. In such a context, the identification and functional characterization of shell matrix components is of major interest. The matrix proteins were initially characterized using a variety of biochemical and molecular methods, which identified at least 10 major eggshell matrix proteins [5,6,8]. The development of high-throughput methods and combined proteomic approaches unexpectedly revealed more than 500 proteins in the eggshell [14-18]. A recent transcriptomic analysis highlighted 605 transcripts which encode 437 proteins over-expressed specifically in the uterus by comparison with 2 other segments of oviduct (magnum and white isthmus), which are not involved in the formation of the shell [19]. The functional characterization or relative role of these proteins in the process of shell formation is still poorly understood.

The prerequisite for shell mineralization in the uterine lumen is the supply of large amounts of calcium (Ca^2+^) and bicarbonate (HCO_3_^−^) in this limited extracellular milieu. Ionized calcium and bicarbonate concentrations in uterine fluid remain supersaturated for the calcite solubility product at the 3 stages of shell formation [20]. Both ions (Ca^2+^, HCO_3_^−^) are supplied by the blood via trans-epithelial transport in the uterus, and require ion channels, ion pumps and ion exchangers. Initial physiological studies described hypothetical mechanisms of mineral secretion (Ca^2+^, HCO_3_^−^) [8,20-22]. Using the transcriptomic data describing uterine gene expression during chicken eggshell calcification [19] and by analogy with mammalian ionic transporters, a general model for ion transfer across the uterine tubular gland cells during eggshell formation suggested the involvement of 31 genes and related proteins [23].

The present study is a complementary approach to functionally characterize the proteins involved in eggshell formation. It aimed to determine a repertoire of functional genes highly expressed in laying hen uterus during shell calcification at the active phase of mineralization when there is a rapid secretion and calcium carbonate deposition around the egg, by comparison of the same tissue in the absence of shell calcification. Eggshell process was suppressed by inducing premature egg expulsion which abolished the uterine egg mechanical stimulation and the calcium demand for shell formation. Consequently, it reduces the blood levels of hormones regulating Ca metabolism during the laying cycle but does not modify the daily pattern of sex steroid hormone involved in ovulation [24,25].

Consequently, this approach allowed us to identify proteins involved in the transport of ionic precursors of the eggshell and the secreted eggshell matrix proteins that regulate the process of mineralization.

## Results

### Uterine genes differentially expressed during the calcification process

We have used the ggallus_ARK_Genomics-20 K oligochips to analyze gene expression in hen uterus during the active phase of shell calcification. Uterine tissues were collected 18 hours post ovulation during shell calcification. This group was compared with uterine tissues harvested at the same stage of egg formation from hens in which the egg had been expelled for 4 consecutive days before eggshell deposition occurs (7–8 hrs post ovulation). The expulsion of the egg prevents the mechanical dilatation of the uterus, the secretion of precursors and the shell mineralization. It was elicited by injection of prostaglandins at least 10 hours before sampling. Such treatment for 4 consecutive days suppressed the stimulation in circulating level of the active metabolite of vitamin D and abolished the expected daily changes in blood ionized calcium but did not affect the plasma level of sex steroids controlling the process of yolk ovulation [24,25]. Consequently, this design focused on genes related to the process of shell formation and to mechanical dilatation induced by the presence of the egg in the uterus. The experimental design used a dye swap procedure (see Methods) of uterine samples collected when a hard shelled egg was calcified in uterus (normal condition, N), or when the uterus was empty due to egg expulsion before onset of calcification (expelled condition, E). Normalization was applied to remove bias due to efficiency of fluorescent dye incorporation. An average of 12,805 spots, representing 63% of the oligos present on the microarrays, were detected and used for statistical analysis (see Methods). The fluorescence ratio, which reflects the relative abundance of mRNA between these two populations for each gene deposited on the microarray was estimated and analyzed. P-values were adjusted by the Benjamini-Hochberg (BH) [26] and Bonferroni (BF) procedures to determine the number of differentially expressed probes (Table 1). BH procedure controls the expected proportion of false positives (False Discovery Rate, FDR), while BF controls the chance of any false positives (Family Wise Error Rate, FWER). The total number of differentially expressed sequences was high when BH procedure was applied (3090 and 1982 at 5 and 1% respectively). When BF adjustment was used, the number of differentially expressed probes was reduced to 694 and 516 at 5% and 1% respectively. Positive N/E ratios corresponded to over-expressed probes when a hard-shelled egg was calcifying in the uterus. Table 1 reports minimum ratio values which reflect the lowest values, and the median ratio values for statistically significant over-expressed probes for the different adjustments and threshold procedures. It is notable that the majority of statistically significant genes revealed with BH test exhibited very low ratios, 50% of them showing an over-expression range of 10 to 40% (N/E ratio from 1.105 to 1.4). 92% of these transcripts showed a differential expression lower than 1.5. In contrast, the BF over-expressed probes corresponded to the highest ratio of N/E and reflected the most over-expressed uterine mRNAs during the shell calcification. The list of over-expressed probes (347) obtained after Bonferroni correction at 1% (BF 0.01), was the one used for later analysis, as this list appeared to be more physiologically relevant.

**Table 1:** Number of differentially expressed probes at the various statistical threshold

**Statistical threshold**	**BH 5%**	**BH 1%**	**BF 5%**	**BF 1%**
**Number of Differentially Expressed (DE) probes**	3090	1982	694	516
**Number of over-expressed probes (N/E)**	1627	1092	438	347
**N/E minimum ratio value**	1.105	1.130	1.255	1.322
**N/E median ratio value**	1.313	1.401	1.719	1.880

The next step was to determine their correspondence with the current chicken mRNA sequences, as annotation provided with our microarrays was obtained with the first draft of the chicken genome assembly (2004) [27]. Consequently, we performed a new annotation of the oligonucleotide sequences deposited on the chip using non-redundant transcript databases (NCBI nr, Gallus gallus Ensembl transcripts) [28,29] and the reference genome (*Gallus gallus* genome, taxon ID: 9031). The ARK_Genomics-20 K oligoarray used in this study is spotted with 20,460 oligonucleotides specific probes to chicken mRNAs (60 mers), with 442 buffers (no template controls) and 218 internal oligonucleotide controls. Amongst the oligonucleotides deposited on the array, 17,631 were found to exhibit a unique match for a single gene, 277 for multiple genes, and 2552 had no correspondences with current nucleotide or genome databases. They consequently were annotated as unknown sequences. Additionally, several redundancies were observed. The most common corresponded to multiple oligonucleotides matching one unique gene product sequence. Taking into account this redundancy, we have determined that the ARK_Genomics-20 K oligoarray contains 14,842 unique genes, representing 57% of genes predicted for *Gallus gallus* (26,028 genes (Entrez Genes Jul 31, 2013)) [30]. Using the update annotations and after removing all redundancies, we determined that the 347 over-expressed probes corresponded to 302 different gene products with annotations (Additional file 1). 296 of them were identified using their corresponding Entrez Gene ID [31], as this database includes nomenclature, Reference Sequences (RefSeqs), maps, pathways, variations, phenotypes, and links to genome-, phenotype-, and locus-specific resources worldwide. The remaining 6 genes were identified using their corresponding Ensembl identifiers. A total of 18 over-expressed probes could not be ascribing to any complete sequence of genes in database (Additional file 1). Consequently, their functional annotation was not possible.

### Verification of level of expression by qRT-PCR analysis

**Figure 1:**
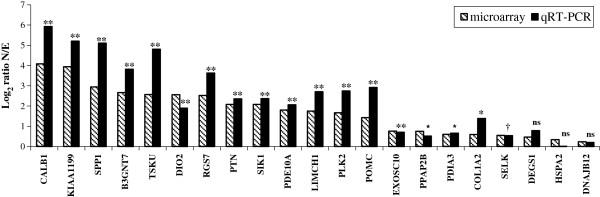
**Comparison of gene expression in the uterus from microarray and qRT-PCR analyses.** Levels of gene expression were expressed as Log2 Ratio N/E. Statistical significance was determined for qRT-PCR expression measurement between normal (N) and expelled conditions (E), as follows: ≤ 0.1 (†), ≤ 0.05 (*), ≤ 0.01 (**), not significant (ns).

Twenty-one over-expressed genes in uterus (shell calcifying) were selected for verification of transcript abundance using quantitative real time RT- PCR on the same biological samples as those used for microarrays (Figure 1). This list is representative of a wide range of gene expression from lowest to highest (1.2 to 17 N/E ratio). Normalized expression levels of genes over-expressed in the uterus with a calcifying shell (Normal condition, N), were compared to that of uterus in which the egg was expelled prior to calcification (Expelled condition, E). N/E ratios determined by quantitative real time RT-PCR (qRT-PCR) are similar to those observed in microarray analyses (Figure 1, Additional file 2). This is confirmed by the high Pearson correlation (0.96) between N/E ratios values from qRT-PCR and microarray. Student t-test performed on the 21 genes quantified by qRT-PCR (Figure 1, Additional file 2) reveals that 18 were over-expressed when a shell was calcifying in the uterus compared to the absence of calcification in the same organ (P < 0.05 and P < 0.1). Three of the differentially expressed genes (DEGS1, HSPA2 and DNAJB12) used for qRT-PCR validation exhibited very low differential expression using microarrays experiments (<1.5). Their differential expression determined using qRT-PCR was also low (1.73, 1.01 and 1.15), but not significantly different between the normal and expelled conditions using qRT-PCR measurement (Additional file 2). Nevertheless, it has to be noted that these 3 genes exhibited similar N/E ratios to the microarray determinations.

### Functional interpretations of the over-expressed genes

Differential analysis and the sequence annotation have determined a repertoire of 302 genes and gene products over-expressed in uterus when eggshell calcification is underway. In order to determine potential functions, genomatix software [32] was used to compare Gene Ontology (GO) terms significantly enriched in the uterus transcriptome during shell calcification, relative to the total GO terms represented in *Gallus gallus.* Eighty seven GO terms (biological process and molecular function), were found to be significantly enriched and were classified in various groups according to their functions (Additional file 3, Figure 2).

**Figure 2:**
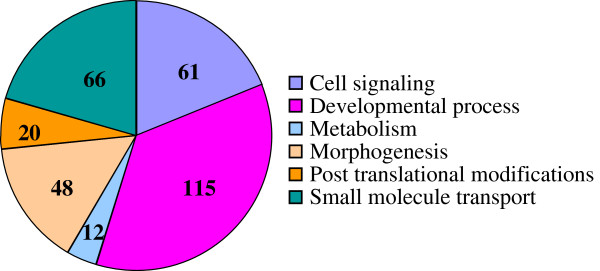
**Piechart diagram showing distribution of over-expressed uterine transcripts when an eggshell calcification is underway.** Gene Ontology (GO) parent terms were used for the overall interpretation of data from microarrays. The numbers of gene products for each GO parent terms are indicated into the diagram.

The largest number of proteins was associated with developmental processes (115 gene products). These proteins are related to biological changes associated with information transfer, growth, and differentiation during the life cycle of organisms, including biology of reproduction as the uterus is a reproductive organ. Another group (morphogenesis, 48 gene products) was also related to developmental biology. Proteins associated with morphogenesis processes control the spatial distribution of cells during the embryonic development of an organism. The other groups were composed of 61 proteins related to cell signaling and trafficking. Additionally, 12 and 20 proteins were related to metabolism and post translational modifications, respectively. Finally a group composed of 66 proteins was associated with the transportation of small molecules (Additional file 3, Figure 2).

Eggshell formation requires highly active mechanisms of ion transfer to secrete the calcium and bicarbonate (carbonate) which are the predominant minerals of the shell. This supply is a prerequisite for shell formation and occurs across the uterine cells. It is notable that amongst the 302 over-expressed genes, 16 encoded uterine proteins that were associated with GO terms related to ionic and calcium transfer or transport (Table 2).

**Table 2:** Proteins associated with ion transport and calcium transfer

**Gene symbol**	**Gene ID**	**Gene description**	**N/E ratio**	**Target ions**
CALB1	396519	Calbindin 1, 28 kDa	16.91	Ca^2+^
ATP2A3	395707	ATPase, Ca++ transporting, ubiquitous	2.97	Ca^2+^
ATP2B1	374244	ATPase, Ca++ transporting, plasma membrane 1	1.95	Ca^2+^
ATP2B2	415958	ATPase, Ca++ transporting, plasma membrane 2	1.82	Ca^2+^
CA2	396257	Carbonic anhydrase II	2.43	HCO3^−^
SLC41A2	427913	Solute carrier family 41, member 2	3.61	Mg^2+^
NIPAL1	428783	NIPA-like domain containing 1	2.64	Mg^2+^
SLC41A3	416033	Solute carrier family 41, member 3	2.64	Mg^2+^
ATP6V1C2	421939	ATPase, H + transporting, lysosomal 42 kDa, V1 subunit C2	2.25	H^+^
KCNH1	421381	Potassium voltage-gated channel, subfamily H (eag-related), member 1	2.75	K^+^
KCNJ2	396328	Potassium inwardly-rectifying channel, subfamily J, member 2	2.16	K^+^
KCNJ16	427812	Potassium inwardly-rectifying channel, subfamily J, member 16	1.75	K^+^
KCNMA1	374065	Potassium large conductance calcium-activated channel, subfamily M, alpha member 1	1.56	K^+^
NKAIN4	419240	Na+/K + transporting ATPase interacting 4	1.92	Na, K^+^
STEAP3	424275	STEAP family member 3	1.85	Fe
SLC25A30	418845	Solute carrier family 25, member 30	1.67	Mitochondrial carrier

### Putative secreted proteins

The 302 over-expressed transcripts in uterus during shell formation can be divided into 2 groups: 1) Intracellular proteins involved in uterine metabolism or in supplying ions, and 2) proteins secreted into the uterine fluid, deposited into the shell, and possibly involved in the biomineralization process. We performed an *in silico* secretomic investigation to determine the over-expressed genes belonging to this second category.

We firstly compared the sequences of the 302 translated proteins arising from our transcriptomic study with those of published eggshell proteomic surveys [14-18]. These proteomic studies used identifiers from 3 different databases. Mann et al. [14,15] described proteins using IPI (International Protein Index database) [33], which was a single database merging ENSEMBL [34] and GeneBank [29]. Numerous changes of protein identifiers have occurred between 2006 (first eggshell proteomic study) and 2011, when this database has closed. The other eggshell proteomic studies used identifiers originating from GeneBank [29], and UniProt (Universal Protein Resource) [35]. To establish correspondence between gene products originating from the diverse annotations, we loaded all protein sequences from these eggshell proteomic studies and used a multi-alignment algorithm to eliminate all redundancies, revealing that the eggshell proteome consists of 538 different proteins. These proteins were compared with gene products from the current transcriptomic study. Forty one genes that are over-expressed in uterus when a shell is forming, encode proteins previously revealed in eggshell by proteomic studies (Additional file 4). It is noteworthy that amongst the remaining proteins, 84 correspond to sequences not present on our microarray and consequently absent from our transcriptomic approach. Forty two additional proteins identified by proteomic approaches were present on the array but not expressed in uterus. These proteins should correspond to proteins originating from other segments of the hen oviduct and passively incorporated into the shell. A total of 21 proteins identified in the shell by proteomic approaches were found to be under-expressed in uterus when a calcifying egg was present, and finally the 350 remaining were present on the microarray but not differentially expressed in the uterus in either physiological condition (Additional file 4).

In a second approach, the 302 proteins derived from the over-expressed genes in uterus during eggshell calcification, were analyzed for the presence of a signal peptide sequence required for protein secretion using a predictor of the classical pathway secretion (SignalP 4.0) [36]. These extracellular proteins are secreted by the endoplasmic reticulum (ER)/Golgi-dependent pathway. However, some of these proteins can be transmembrane proteins with extracellular domains, and are not secreted in the uterine fluid. They were determined using TMHMM2.0 [37] and only proteins with a signal peptide and not reported as transmembrane proteins were selected as secreted. Further analyse based on secretion signals and functional annotations were performed using YLOC [38-40] to determine additional genes encoding uterine proteins secreted by a non-classical way, in absence of a signal peptide. Altogether, we determined that 58 proteins are both potentially secreted and over-expressed in uterus during eggshell calcification (Table 3). Twenty-two of them were present in the shell proteome and 33 are newly identified from the current transcriptomic data (Table 3). Additionally, 2 over-expressed genes in the uterus were not predicted to be secreted using *in silico* tools, but were reported in proteomic surveys and annotated as secreted in databases. They could correspond to proteins secreted by non-classical ways (Table 3).

**Table 3:** Functional annotations of putative secreted uterine proteins that are up-regulated when a hard-shelled egg is present

**Potential functions**	**Gene symbol (Gene Id)**	**Gene description**
**Mineralization Interactions with calcium**	SPP1* (395210)	Secreted phosphoprotein 1 (osteopontin), involved in bone mineralization and present in chicken eggshell
	PENK* (421131)	Proenkephalin
	COL1A2* (396243)	Collagen, type I, alpha 2
	DCN** (417892)	Decorin, leucine-rich repeats (LRRs) participate in protein-protein interactions
	SPARCL1** (422586)	SPARC-like 1 (hevin), extracellular Ca2+ binding domain (containing 2 EF-hand motifs), serine protease inhibitor
	CD34** (419856)	CD34 molecule, podocalyxin, sialoprotein highly negatively charged
	CRELD2** (417735)	Cysteine-rich with calcium binding EGF-like domains 2
	MCFD2** (421413)	Multiple coagulation factor deficiency 2, contains calcium-binding EF-hand
	MATN2*** (426584)	Matrilin 2, calcium-binding EGF-like domain, mediate cell-matrix and matrix-matrix interactions in cartilaginous
	SLIT2* (373967)	Slit homolog 2 (Drosophila), developmental biology with EGF domains includng calcium binding domains
**Proteoglycans**	HS6ST2* (395150)	Heparan sulfate 6-O-sulfotransferase 2
	EXT1*** (420283)	Exostosin 1, involved in glycosaminoglycans biosynthesis
	MGAT4B** (416285)	Mannosyl (alpha-1,3-)-glycoprotein beta-1,4-N-acetylglucosaminyltransferase, isozyme B, N-Glycan biosynthesis
	TSKU* (419088)	Tsukushi small leucine rich proteoglycan homolog, protein-protein interactions
	GPC1* (424770)	Glypican 1
	ADAMTS1** (418479)	ADAM metallopeptidase with thrombospondin type 1 motif, 1
**Molecular chaperone**	CLU* (395722)	Clusterin, molecular chaperone
	GKN2-OCX21* (419515)	Gastrokine 2 (ovocalyxin-21), eggshell specific protein, brichos domain, molecular chaperone
	HSPA5* (396487)	Heat shock 70 kDa protein 5 (glucose-regulated protein, 78 kDa), chaperone help to fold many proteins
	HSPA13** (418469)	Heat shock protein 70 kDa family, member 13, molecular chaperone
	HSP90B1** (374163)	Endoplasmin, heat shock protein 90 kDa beta (Grp94), member 1, molecular chaperone
	HYOU1* (428251)	Hypoxia up-regulated 1, HSP protein, molecular chaperone
	ERLEC1* (421220)	Endoplasmic reticulum lectin 1
	DNAJB9** (417783)	DnaJ/Hsp40 (heat shock protein 40) proteins, crucial roles in protein translation, folding, unfolding, translocation, and degradation, molecular chaperone
	FICD** (416889)	FIC domain containing tetratricopeptide repeat domain involved in variety of functions including protein-protein interactions, molecular chaperone
	GRIP2** (416043)	Glutamate receptor interacting protein 2, involved in protein-protein interactions
**Antimicrobial proteins**	OCX36* (419289)	Ovocalyxin-36, LBP/BPI protein, antimicrobial
	BPIL3** (419290)	Bactericidal/permeability-increasing protein-like 3, LBP/BPI protein, antimicrobial
	LY86* (420872)	Lymphocyte antigen 86, involved in innate immune response and binding to bacterial LPS
	PTN* (418125)	Pleiotrophin, heparin binding protein, antimicrobial
	SEMA3C* (374090)	Sema domain, immunoglobulin domain (Ig), short basic domain, secreted, (semaphorin) 3C, developmental biology
	LOC422316** (422316)	Vascular endothelial growth factor receptor kdr-like, IG-like domain
**SERPIN**	PROC** (395085)	Protein C (inactivator of coagulation factors), trypsin-like serine protease with EGF-like calcium binding domain
**Other roles**	P4HA1** (423704)	Prolyl 4-hydroxylase, alpha polypeptide I
	SPON1* (395657)	Spondin 1, extracellular matrix protein involved in patterning axonal growth trajectory
	POMC* (422011)	Proopiomelanocortin
	SEMA6D* (415430)	Sema domain, transmembrane domain (TM), and cytoplasmic domain, (semaphorin) 6D, involved in the development of the nervous system and in axonal guidance
	P4HB* (374091)	Prolyl 4-hydroxylase, beta polypeptide, containing thiredoxin domain
	PRDX4* (418601)	Peroxiredoxin 4
	FAM3C* (417758)	Family with sequence similarity 3, member C
	LOC776741** (776741)	Uncharacterized LOC776741
	RET** (396107)	Ret proto-oncogene, cadherin repeat-like domain involved in cell-cell contact when bound to calcium
	GREM1** (395826)	Gremlin 1, extracellular DAN domain
	WNT11** (395562)	Wingless-type MMTV integration site family, member 11
	ROS1** (396192)	C-ros oncogene 1 receptor tyrosine kinase, contains Fibronectin type 3 and protein Tyrosine Kinase domains
	ERP29** (416882)	Endoplasmic reticulum protein 29, thioredoxin domain, oxidoreductase
	COMT** (416783)	Catechol-O-methyltransferase
	CCDC80** (395074)	Coiled-coil domain containing 80 involved in cell adhesion;
	PDGFD** (418978)	Platelet derived growth factor D, biology of development
	ERO1LB** (421509)	ERO1-like beta (S. cerevisiae)
	PDIA6** (421940)	Protein disulfide isomerase family A, member 6, contains thioredoxin domain
	MANF** (770664)	Mesencephalic astrocyte-derived neurotrophic factor, RNA binding protein
	CYP26A1** (408183)	Cytochrome P450, family 26, subfamily A, polypeptide 1 involved in the oxidative degradation of various compound
	AGR2** (420596)	Anterior gradient homolog 2 (Xenopus laevis), containing Thiroedoxin domain
	TNFRSF6B** (395096)	Tumor necrosis factor receptor superfamily domain, involved in nvolved in inflammation response, apoptosis, autoimmunity and organogenesis
	PCDH19** (422263)	Protocadherin 19, Cadherin tandem repeat domain involved in Ca2 + −mediated cell-cell adhesion
	PDIA3 ** (373899)	Protein disulfide isomerase family A, member 3, containing thiroedoxin domain

*Predicted to be secreted and already identified in the eggshell.

**Predicted to be secreted but not previously identified in the eggshell.

***Proteins already identified in the eggshell, but not predicted to be secreted.

## Discussion

The chicken eggshell is a highly ordered structure with exceptional mechanical properties [8]. It is 95% calcium carbonate in its calcitic polymorph and 3.5% organic macromolecules. Its formation depends upon numerous physiological adaptations and processes by the uterine cells, which exhibit the capacity to transfer large amounts of Ca^2+^ and HCO_3_^−^[22,23]. The calcium and bicarbonate ions, and precursors of the organic constituents, are secreted into the acellular uterine fluid where they interact to form the complex bioceramic eggshell [5,7,8]. This mechanism is known as controlled biomineralization, and is widely represented by various organisms (corals, molluscs, birds…); it occurs by direct interactions between organic and mineral phases with no direct actions of cells [12,13,41]. Chicken eggshell mineralization occurs in 3 distinct phases (initiation, growth and terminal) [20]. The uterus secretes the mineral and proteins as soluble precursors into the acellular uterine fluid. The shell calcification is initiated by nucleation upon a fibrous scaffold (mammillary knobs on eggshell membranes). The growing crystals interact with the shell organic matrix to form a compact layer with highly ordered microstructure and texture resulting in an eggshell with exceptional mechanical properties [4,6-8]. There is an adaptation of the protein contents at each phase of shell formation in the uterine fluid [10], and the calcification process is the result of direct interactions of organic matrix proteins and minerals comprising the milieu [4,6,8]. Recent high-throughput studies allowed the identification of more than 500 proteins in the shell [14-18], and the identification of 469 uterine specific transcripts [19]. These major advances led to a global identification of the molecular actors potentially involved in the shell calcification process.

In the present study, we used a transcriptomic approach to identify and characterize proteins potentially involved in the supply of minerals and in the mechanisms of avian shell mineralization. In a previous study, the calcifying uterus was compared to 2 other segments of the oviduct not involved in shell formation (magnum and white isthmus secreting egg white and eggshell membranes respectively), which demonstrated that the uterine transcriptome in this specific physiological state consisted of 605 differentially expressed genes [19]. In the current study, we have compared gene expression in the uterus collected during the active calcification phase (18 hours post ovulation), when there is a rapid secretion and growth of CaCO_3_ leading to the formation of the compact shell layer *versus* uterus in the absence of egg and eggshell formation. Egg expulsion was induced 10 hours before sampling of the tissue by injecting prostaglandins which are involved in the physiological process of egg oviposition. Previous reports showed that this treatment did not affected the daily pattern of sex steroid but egg expulsion abolished the changes in ionized calcium and in plasma level of 1,25 (OH)2 D3 observed during the period of shell formation [25]. During the daily laying cycle, the genes over-expressed during the active calcification phase are stimulated either by the reproductive hormones associated with egg formation and yolk ovulation, those regulating the calcium metabolism, or the mechanical constraint due to the presence of the egg in the uterus. Egg expulsion suppresses both the mechanical stress and the Ca demand but does not affect the reproductive hormone cycle [24,25]. The change in uterine physiology was elicited by premature expulsion of the egg, before shell formation, for 3–4 consecutive days. The early egg expulsion eliminates the calcium and bicarbonate requirement for shell formation, as well as mechanical stimulation of the uterine wall due to egg entry. This experimental approach is complementary to the sampling of hen uterus at an early stage of egg formation when the egg is present in the magnum (albumen secretion) or at a later stage when the calcification takes place in the uterus as used in the study on regulation of uterine calbindin [42,43] or when exploring the temporal regulation of numerous gene in the oviduct [44]. Both experimental approaches take into account the mechanical stimulation of uterus and the presence of shell secretion but the egg expulsion model largely affected the Ca metabolism. It should reveal up-regulated genes either associated with the process of biomineralization and the supply of shell precursors in particular those coding for ionic transport proteins in addition to those stimulated by mechanical stretching as already reported for osteopontin and glypican-4 when exploring the change during the laying cycle [45,46]. Our approach identified a cluster of 302 genes encoding uterine proteins over-expressed during shell calcification. Over-expressed genes were defined using a Bonferroni correction at a threshold of 1%, with over-expression ranging from 32% to 1591% (N/E ratio from 1.32 to 16.91, with a median value of 1.88; Table 1).

**Figure 3:**
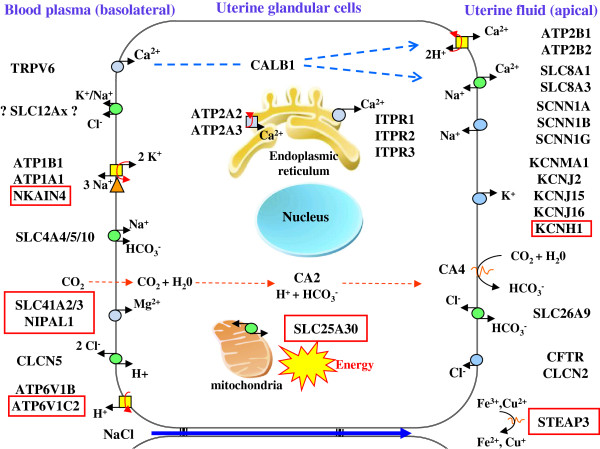
**General model describing uterine ion transporters during eggshell calcification **[23]**, and updated with novel ion transporters identified in this study.** Red squared indicated novel proteins identified in this study.

Ontology term enrichment analysis (Biological Process and Molecular Functions GO terms) of the 302 over-expressed uterine transcripts showed that the most over-represented proteins are related to reproductive function (developmental processes, morphogenesis…) (Additional file 3, Figure 2) in agreement with the observation of Jeong et al. [44]. This enrichment is in accordance with the function of the hen oviduct in chicken reproduction. In addition, we paid particular attention to genes encoding proteins potentially involved in shell mineralization taking into account that our model largely affected Ca metabolism. From the total list of genes over-expressed in uterus during eggshell calcification, as expected, Gene Ontology and chicken gene nomenclature [47,48], identified 16 proteins related to ion transport and ion transfer (Table 2). The current study identified novel candidates potentially involved in ion transfer by uterine cells relative to our previous study and model [23], and allowed an update of the earlier model (Figure 3).

Calcium is an essential element in the calcification process of the eggshell; it is not stored in uterus but is continuously supplied from the blood. Hen uterine glandular cells must transfer large amounts of calcium into the uterine lumen against the concentration gradient while preserving a low level of intracellular calcium (<0.0002 mM). As previously proposed, calcium transfer into the uterine fluid involves TRPV6 or other Ca channels for the entry in the cell, calbindin D28 k (CALB1) for the intracellular transport and the calcium output is ensured by Na^+^ /Ca^2+^ or Ca^2+^ /2H^+^ Calcium exchangers [23] (Figure 3). We observed that calbindin D28K (CALB1) was highly over-expressed in our model during calcification (16.91, Table 2) in agreement with previous observation [23,42-44]. This observation is consistent with its described role associated with Ca^2+^ transport, and its cell protective effect against high concentrations of Ca^2+^ or apoptotic cellular degradation [49]. Low free calcium levels in the cell are maintained by calcium uptake in the endoplasmic reticulum *via* ATP depending calcium pumps (Figure 3). Our microarray analysis showed an over-expression of ATP2A3 (N/E ratio of 2.97), when the egg is being calcified (Table 2). Finally, the calcium pumps ATP2B1 and ATP2B2, which can extrude calcium from the cytosol to the extracellular against a strong electrochemical gradient [50] were over-expressed in our study (1.95 and 1.82 respectively; Table 2) in agreement with our previous study [23], suggesting their involvement in the secretion of calcium into the uterine lumen.

Carbonate is another major component of the shell. Previous studies have shown that the CO_3_^2−^ of the eggshell is not derived from the blood HCO_3_^−^ but rather from the plasma CO_2_, which is hydrated in the uterus to produce bicarbonate [51]. Carbonic anhydrases (CA) are responsible for the transformation of CO_2_ to HCO_3_^−^ and histochemical studies have implicated the uterine glandular cells as the site of the carbonic anhydrase activity in hen shell gland [52,53]. In mammals, intracellular carbonic anhydrases include five cytosolic isoforms (I, II, III, VII and XIII) [54]. In *Gallus gallus*, only the cytosolic CA 2, 3 and 7 are referenced in the Gene database (NCBI, Ensembl). We have observed in our study a very high level of CA2 expression and no expression of CA3 and CA7. CA2 appears to play a pivotal role for conversion of intracellular CO_2_ to HCO_3_^−^ in chickens.

The transfer of calcium and bicarbonate from the blood to the uterine fluid involves additional ion transfer to maintain cell homeostasis. There are exchanges of Na^+^, K^+^, Mg^2+^ and Cl^−^ to maintain physiological ionic concentrations in the cell. Na^+^ is absorbed from the uterine fluid by Na^+^/Ca^2+^ exchangers and by amiloride-sensitive Na^+^ channels at the apical membrane (Figure 3) and is extruded from the cell at the basolateral membrane against the electrochemical gradient by the Na^+^/K^+^ ATPase which transports Na^+^ out and K^+^ into the cells [23]. Indeed, potassium is secreted into the uterine fluid during the calcification of the shell and its concentration varies in this milieu from 12 mM at the initial phase of calcification and increases to 60 mM during the active growth phase [22]. Over-expression of the α1 and β1 subunits of the Na/K ATPase (ATP1A1 and B1) has been demonstrated in the uterus of laying hens during shell calcification [23,55] but was not statistically different in our study (N/E ratio 1.5). In contrast, we observed the over-expression (N/E ratio 1.92; Table 2), of NKAIN4, belonging to the NKAIN family. One member of this family (NKAIN1) interacts with the β1 subunit (ATP1B1) of the Na/K ATPase in mouse and drosophila [56], and consequently, we have linked NKAIN4 to the influx of potassium (Figure 3). The passive diffusion of potassium to the uterine lumen through the apical membrane is induced by a high concentration of intracellular potassium in cells (>100 mM). Five gene products in connection with the transfer of potassium, during its secretion into the uterine lumen, were detected in this study (Table 2, Figure 3) and confirmed previous observations of over-expression of two of these voltage-dependent ion channels regulated by the extracellular K ^+^ (KCNJ2, KCNJ16; Table 2 and Figure 3) and of the alpha subunit 1 of large conductance calcium activated potassium channel (KCNMA1) [23]. Their activation would modulate the export of potassium thus contributing to the membrane repolarization. A recent study has also demonstrated the role of the KCNMA1 channel in the contraction of smooth muscle cells in the uterine myometrium of pregnant women [57]. In addition, we observed the over-expression of a novel gene of the voltage-dependent ion channel family, KCNH1 (ratio 2.75) during shell calcification (Table 2) consistent with its involvement in potassium efflux from the cell. This gene is known to be expressed in mouse heart, brain and myometrium, and to play a role in the suppression of contractile activity of the uterus [58].

During eggshell formation, a progressive acidification of uterine fluid and of the uterine glandular cells occurs. Two H^+^ ions are produced for each CO_3_^2−^ formed [20,22]. V-type H + ATPases (VAT) allow transfer of H^+^ by hydrolysis of ATP [59]. Our previous study showed the over-expression of VAT subunit B (ATP6V1B2) in the hen uterus suggesting its participation in proton transfer from the chicken uterine cell to plasma [23]. We report here the over-expression of an additional V-ATPase subunit, the H + transporting lysosomal 42 kDa, V1 subunit C2 (ATP6V1C2) which is 2.25-fold over-expressed during the calcification process. The localization of these proteins in the basolateral membrane is likely because of the acidification of plasma during calcification and the absence of other transporters but should be confirmed by immunohistochemistry.

The eggshell contains a small amount of magnesium (around 0.35%) which slightly increases with augmented supply of dietary Mg [60]. It is therefore likely that the uterus secretes Mg ion. Our study showed over-expression of two of the three members identified to date of the MgtE-like magnesium transporter family (SLC41), SLC41A2 and SCL41A3, with fold changes of 3.61 and 2.64, respectively (Table 2), These transporters are therefore present in the chicken uterine cell and were included in the general model describing uterine ion transporters during eggshell calcification (Figure 3). SLC41A2 gene is a carrier of magnesium associated with the plasma membrane that mediates Mg^2+^ influx [61,62]. The direction of Mg^2+^ flux for SLC41A3 is unknown [63] but the favorable gradient between plasma and intracellular Mg support a role in cell influx [64]. We also observed an over-expression of NIPAL1, also known as magnesium transporter NIPA3 [65]. Expression of additional Mg^2+^ transporters was observed in the uterus but without over-expression during calcification (MAGT1, MMGT1, NIPA1-2, NIPAL3). The respective roles of all these carriers in Mg^2+^ secretion and in magnesium homeostasis in the uterine cells remain largely undetermined.

Iron and copper are also present in eggshell at low concentrations and are essential elements for cell metabolism in particular for cytochromes oxidases in the mitochondrial respiratory chain [66,67]. STEAP3 is over-expressed in the uterus during calcification (fold change of 1.85) and might participate in iron homeostasis of the uterine cells as the six-transmembrane epithelial antigen of prostate 3 (STEAP3) are known to reduce Fe^3+^ to Fe^2+^, and Cu^2+^ to Cu^+^[67,68]. Finally, the over-expressed SLC25A30 is a mitochondrial carrier, also known as KMCP1. This carrier has been proposed to be involved in mitochondrial metabolism when stimulated, in particular when the cellular redox balance tends toward a pro-oxidant status [69]. It might transport substrates which widely vary in their structure and size from the smallest, H+, to the largest and most highly charged species, ATP [69,70].

The main function of the chicken uterus is daily calcification of the shell during the 19 hours period while the egg remains in this organ. The growing crystals interact with the shell organic matrix to form a compact layer with highly ordered microstructure and texture resulting in an eggshell with exceptional mechanical properties [4,6-8]. In order to gain insight into the functional properties of the matrix proteins in eggshell mineralization, we have performed a secretomic inventory of the proteins over-expressed in the uterus when shell formation takes place. This group of proteins was explored by sorting from the over-expressed genes, the proteins secreted in the external milieu (uterine fluid) where calcification takes place. Two complementary strategies were applied. Firstly, the proteins encoded by the 302 over-expressed genes in uterus when a shell was under formation, were compared with the 538 proteins already identified in the shell by proteomic approaches [14-18]. This analysis indicated that 76% of the already identified proteins in the shell were expressed by the uterus but only 41 (7.3%) were differentially expressed when the active growth phase occurred (Additional file 4). It has also to be noted that 42 of the proteins already identified in the shell were not expressed in the uterus. This observation confirmed the hypothesis that the eggshell proteome revealed proteins derived from degraded cells or basement membranes or issued from the upper oviduct segments and incorporated passively into the shell as already suggested [14,71]. Secondly, the presence of a signal peptide on their N-terminal amino acid sequence were explored in the protein sequence issued from the list of over-expressed genes to determine which proteins are potentially secreted by uterine cells and are stimulated during the calcification phase in the uterus. The presence of this sequence is observed for proteins secreted through the classical endoplasmic reticulum/Golgi apparatus. Some transmembrane proteins presented signal peptides but are not present as free proteins in the external milieu. These particular proteins were determined using transmembrane predictors and were removed to constitute the final list of excreted proteins. In addition there are secretory proteins which do not have a signal peptide [72]. Additional datamining procedures were applied to identify these proteins using non classical secretory pathways. Table 3 showed the proteins over-expressed in uterus and predicted as being secreted in the uterine fluid. We have investigated the potential functions of these 57 proteins using annotations in databases, motifs and domains databases (Table 3). These proteins were classified according to their biological functions in the egg.

**Figure 4:**
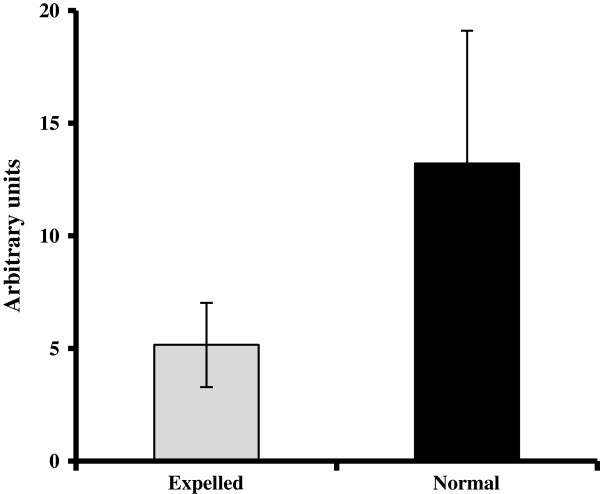
Ovocleidin-116 mRNA expression pattern in normal and expelled conditions detected in uterus using qRT-PCR.

The first group corresponded to proteins potentially involved in biomineralization. Numerous studies have demonstrated the importance of the interactions between these proteins and the crystal formation [8]. The secreted phosphoprotein 1 (SPP1, osteopontin) is an acidic protein, glycosylated, highly phosphorylated and associated with calcium metabolism in birds and mammals. In chicken, osteopontin is found in both bone and eggshell [73,74] and is localized in palisade and mammillary layers of the shell [75]. Its expression is stimulated by mechanical distension of the uterine wall the presence of the egg in the uterus [44,46]. SPP1 is an inhibitor of calcium carbonate precipitation [76], interacting with specific eggshell calcite faces [75]. Ovocleidin-116 (OC-116) is the first eggshell matrix to be cloned and is the most abundant eggshell matrix protein, abundant in uterine fluid during the active phase of shell calcification [77,78]. It is also present in the bone with a role similar to its mammalian ortholog MEPE [79], and consequently is suspected to play a crucial role in the shell mineralization process. OC-116 was present on the array, but its expression was saturated, and consequently its N/E ratio could not be determined. To overcome this problem, we designed specific primers to determine its expression using quantitative RT-PCR in both normal and expelled tissues (Figure 4). The relative expression of OC-116 was 2.6 times higher in normal uterine tissues compared to expelled (p < 0.01).

This uterine gene expression profiling study also highlights other proteins known to be mainly involved in the mineralization of bone and cartilage. The proenkephalin-A (PENK) was largely over expressed as reported by Jeong et al. [44]. This protein and its derived peptides cause an inhibition of the activity of alkaline phosphatase in mammals [80]. Collagen type 1 alpha 2 (COL1A2) is a matrix protein extracellular fibrils forming the tendons and ligaments. Decorin (DCN) is a protein of the connective tissues playing a role in the assembly of the matrix by linking the type I and II collagen fibrils [81]. This protein is regulated by the extracellular matrix Hevin (SPARCL1) which possesses calcium binding domains [82]. Another interesting protein is podocalyxin (CD34), a sialoprotein already identified in previous transcriptomic study [19], and suspected to interact with calcium because of its high negative charge. Calcium binding proteins interact with calcium to favor crystal nucleation or drive morphology of crystals by interacting with particular faces. In the present study, we reported the presence of CRELD2, MCFD2, MATN2 and SLIT2, 3 additional proteins which contains EGF-like and EF-hand calcium binding domains. The presence of SLIT2 was already reported in our previous study on uterine secretome [19].

Proteoglycans are major actors of calcification in biomineralization process and have been reported to be abundant in the chicken eggshell [83,84]. These compounds are the result of the combination of a protein core with negatively charged complex polysaccharides. They can consequently interact with calcium and are suspected to play a major role in the process of shell mineralization. Amongst secretory proteins (Table 3), many of them are related to the biosynthesis of glycans and proteoglycans. This is the case of the heparin sulphate 6-0-sulfotransferase (HS6ST2) and of exostosin 1 (EXT1). Other candidates are the alpha-1,3-mannosyl-glycoprotein beta 1-4-N-acetylglucosaminyltransferase B (MGAT4B), a glycosyltransferase involved in the glycan biosynthesis. Ttsukushin (TSKU) belongs to the family of proteoglycan proteins containing small areas leucine-rich repeat (SLRP). These proteins have a key role in coordinating multiple extracellular signaling pathways during embryonic development of chicken, frog and mouse [85]. Glypican 1 (GPC1) is a glycoprotein belonging to the heparan sulfate proteoglycans of the extracellular matrix-related plasma membrane [86]. Disintegrin and metalloproteinase with thrombospondin motifs 1 (ADAMTS1) are peptidases involved in the degradation of chondroitin sulfate proteoglycan and in the renewal of the extracellular matrix [87].

The regulation of protein activities of eggshell matrix *in situ* relies on direct action of proteins to inhibit or activate the molecular actors present in the milieu. Proteins involved in protein-protein interactions that might be involved in the proper folding of the eggshell matrix might be involved in this regulation process. This study revealed several molecular chaperones. Clusterin (CLU) is a chaperone protein of the uterine fluid, suspected to prevent the premature aggregation and precipitation of eggshell matrix components before and during their assembly into the rigid protein scaffold necessary for ordered mineralization [88]. Ovocalyxin-21 (GKN2) is an eggshell specific matrix protein containing a brichos domain associated to molecular chaperone [5]. This study identified 11 additional proteins annotated as chaperones or involved in protein-protein interactions (Table 3). This is the case for 2 heat shock proteins HSPA5 and −13. Another heat shock protein (HSP90B1) was previously identified in the chicken shell gland as endoplamin [19]. A role of molecular chaperone can also be ascribed to a protein over-expressed in condition of hypoxia (HYOU1), to the lectine1 endoplasmic reticulum (ERLEC1), to the DnaJ homolog subfamily B member 9 (DNAJB9) and to FICD. Additionally, the proteoglycan homolog (TSKU), the glutamate receptor interacting protein (GRIP2), decorin (DCN), and matrilin 2 (MATN2) are proteins known to play a role in protein-protein interactions and consequently could be involved in assembly and folding of the eggshell matrix.

The shell is a physically protective barrier for the developing embryo against microbial invasion but surprisingly, this dry mineral material also contains proteins with antimicrobial properties. The egg chemical protection is most commonly ascribed to the liquid egg-white that possesses numerous proteins responsible for its antimicrobial properties [2]. The eggshell is indeed not a suitable medium for microbial growth, due to its physical characteristics (solid structure with low moisture content). Antimicrobial proteins secreted into the uterine fluid might keep the lumen free of bacteria and would contribute to the protection of the forming egg. Additionally, the dissolution of the calcium from the base of the eggshell for bone embryonic development might solubilize shell antimicrobial components and contribute to protection of the embryo at the end of its development. Several antimicrobial proteins were over-expressed in uterus during shell calcification including BPIL3 and ovocalyxin-36 (Table 3). Ovocalyxin-36 (BPIFB8) is an eggshell matrix protein highly secreted by uterine glandular cells during the active calcification phase and is present in the eggshell membranes and egg vitelline membrane [89]. Ovocalyxin-36 and BPIL3 belongs to lipopolysaccharide-binding proteins and Bactericidal Permeability Increasing (BPI) family which is well known in mammals for their involvement in defense against bacteria. These proteins belong to the BPI fold superfamily containing PLUNC/PSP/BSP30/SMGB [90]. Members of this family bind to the lipid A portion of lipopolysaccharide cell wall in Gram negative bacteria, leading to the death of bacteria. Other proteins reported in this study may also have a role as antibacterial proteins as suggested by their affinity for bacterial membrane components. Lymphocyte antigen 86 (LY86) interact with bacterial LPS [91]. Pleiothrophin (PTN) is an heparin binding protein and antimicrobial activities were recently demonstrated for egg protein binding heparin [92]. It has also to be noted that the list of secreted over-expressed proteins in the uterus (Table 3), contained SEMAC3C and LOC422316, 2 proteins with immunoglobulin-like domains potentially related to the innate immune defense.

The last group of functional candidates over-expressed during calcification corresponded to proteases and antiproteases. They might contribute during the calcification process, either by degrading proteins or by regulating maturation of proteins. Proteolytic activity present in uterine fluid varies according to the stage of the calcification [93]. This study also identified ADAMTS1, a metallopeptidase with thrombospondin motif, and SPARCL1 and PROC as 2 Serine Protease Inhibitors (SERPIN).

## Conclusions

The uterus secretes a huge amount of calcium and bicarbonate to calcify the eggshell and to provide the shell matrix proteins which control its mineralization in an acellular milieu. Comparison of global gene expression in the presence or absence of shell formation is a powerful model to reveal candidate proteins involved in both processes of mineral secretion and shell mineralization. Transcriptome analysis of the uterus reveals 302 over-expressed genes when shell calcification takes places. A large number were classified by gene ontology as being involved in development, information transfer, metabolism of cells and tissue. The term “biomineralization” is not identified because of the specificity of birds proteins involved in this process and the absence of mammal homologues. In contrast, 16 gene products encoding proteins associated with mineral supply and 57 proteins secreted in the uterine fluid and potentially involved in the calcification process were reported. Identification of novel ionic transporters has been used to develop a model describing additional steps of ion transfers through the glandular cells of the uterus to actively secrete calcium and bicarbonate while maintaining cell homeostasis. The exploration of secreted proteins showed that a majority were present in the eggshell but represented less than 10% of total proteins identified in the shell matrix. They can be mainly classified into five functions; mineralization, proteoglycans formation, chaperone proteins, proteases/antiproteases and antibacterial proteins. This study completes the screening of candidate proteins likely to be involved in shell formation and gives some insight into their putative function. This information is a prerequisite for developing more quantitative studies *in vivo* (genetic, physiological studies combined with quantification of uterine gene expression and proteins abundance) or *in vitro* (calcite growth in presence of candidate proteins) to identify the prominent molecules controlling shell formation.

## Methods

### Ethical statement, animals handling and housing

All experiments, including all animal-handling protocols, were carried out in accordance with the European Communities Council Directives concerning the practice for the care and Use of Animals for Scientific purposes and the French ministerial on Animal experimentation under the supervision of authorized scientists (authorization # 7323, delivered by the DDPP, direction départementale de la protection des populations, d’Indre et Loire). The experimental unit UE-PEAT 1295 where the birds were kept holds a permit for rearing birds and for the euthanasia of experimental animals (decree N° B37-175-1 of August 28th 2012 delivered by the Préfecture d’Indre et Loire following the inspection of the Department Direction of Veterinary Services). The protocol (00159.02) was approved by an ethical committee (comité d’éthique de Val de Loire, officially registered under number 19 of the French national ethic committee for animal experimentation).

Forty two brown egg-laying hens (ISA Brown strain) were caged individually and subjected to a light/dark cycle of 14 h light and 10 h darkness. Hens were fed a layer mash as recommended by the Institut National de la Recherche Agronomique (INRA). Each cage was equipped with a device for automatic recording of oviposition time (time of egg expulsion).

### Collection of uterine tissues

Uterine tissues were harvested from 8 birds during the rapid growth phase of calcification (18 ± 1 hours post ovulation, Normal group; N). Additionally, uterine tissues were also collected from 8 birds in which 50 μg of prostaglandin 2α was injected during 4 consecutive days 7 to 8 hours after ovulation, to expel the egg before mineralization has begun (Expelled group: E). Uterine tissues were scraped and 100 mg were weighed in individual replicates before being immersed in RNA preservatives (RNAlater, Applied Biosytems/Ambion, Austin, USA) and stored at −80°C.

### RNA preparation, microarray hybridization and raw data acquisition

The ggallus_ARK-Genomics_20K (GEO: GPL6049) microarray contains 20,460 oligonucleotides (60–75 bases). DNA chips were produced by the Centre de Ressources Biologiques GADIE (INRA Jouy en Josas) [94], and contained the original oligonucleotides series, 442 buffers and 218 internal control oligonucleotides (operon). Total RNA was extracted from frozen uterine tissue samples using a commercial kit and simultaneously treated with DNase according to the manufacturer’s procedure (NucleoSpin RNA Midi, Machererey-Nagel, Hoerd, France). RNA concentrations were measured at 260 nm. The integrity of RNA was evaluated on a 1% agarose gel and with an Agilent 2100 Bioanalyzer (Agilent Technologies, Massy, France). Only RNA samples with a 28S/18S ratio > 1.3 were used for labeling and hybridization.

Sixty micrograms of total RNA were used for indirect cDNA labeling with Alexa fluorescent probes using SuperScript™ Indirect cDNA Labeling (Invitrogen, Cergy-Pontoise, France) according to the manufacturer’s instructions. A dye-swap design was used. Normal (N) and Expelled (E) uterine tissues were labelled subsequently with both Alexa 647 and −555 fluorescent dyes (Molecular Probes, Invitrogen). A total of 16 microarray slides were used for hybridization. The dye incorporation of purified labelled cDNA samples were quantified using a NanoDrop spectrophotometer (ND 1000, Thermo Fisher Scientific, Illkirch, France).

An equal amount (fifty to sixty pmoles) of Alexa 555 and −647 cDNA probes (1 pmol/μl) corresponding to each N and E contrasts, was mixed with 50 to 60 μL of PRONTO buffer (Corning, Life Sciences), then hybridized at 42°C for 16 h using a dynamic Slide-Booster system (Olympus Advalytix, Germany). Microarrays were then washed with the AdvaWash apparatus (Olympus Advalytix, Germany). Washing was performed according to the PRONTO kit manufacturer’s procedure.

The quantitative measurement of fluorescent signals was performed using a GenePix 4000B laser scan (Molecular Devices, St. Grégoire, FRANCE). Microarray slides were scanned at 532 nm for Alexa 555 and 635 for Alexa 647. Analysis of fluorescent spots was performed with the GenePixPro 6.0 software. It was used to acquire the fluorescence, filter the expressed and non-expressed spots, align the spots, quantify their intensity and finally export GenePix report (GPR) files containing spot intensity raw data. The GPR files were stored in the BioArray Software Environment (BASE) of SIGENAE (Système d’Information du projet d’Analyse des Genomes des Animaux d’Elevage) for further processing. Spots with a lack of homogeneity of the fluorescence or overlapping with neighbouring spots were manually rejected.

### Microarrays statistical data analysis

Gene expression was compared between uterine tissues in normal (N) and expelled conditions (E). Differentially expressed genes were determined using the Limma package developed for R [95] allowing standardization and data analysis of differential expression between different biological conditions [96]. A preliminary processing of data removed non-expressed spots and those corresponding to buffer and operon controls. Spots intensities were retained when present in at least 50% of the samples. Spot intensities were calculated using median values which were transformed to log2 values. Log2 median ratio values were normalized assuming that the majority of expressed genes did not differ between contrasts (Normal and expelled conditions). Normalization consisted of a local polynomial regression fitting (Loess) to remove dye bias due to the incorporation of fluorescence. Spots differentially expressed between conditions N and E were determined with a moderated t-test, followed by two procedures for multiple testing corrections (Bonferroni method (BF) and Benjamini and Hochberg (BH)) [26], to control the Family Wise Error Rate (FWER) and false discovery rate respectively (5% or 1%).

### Bioinformatic analysis

A new annotation of genes spotted on the microarray was updated as the information provided with slides was established using the first draft of the chicken genome assembly in 2004. NCBI megablast [97] was used against non-redundant transcript databases (NCBI nr, Ensembl transcripts), and reference genome (Gallus_gallus-2.1, May 2006 assembly) [27] for *Gallus gallus* species (Taxid: 9031). Annotations were then determined from identical transcripts and/or location on genome using the NCBI E-utilities (databases: Unigene, Gene, Nucleotide) [97] and Ensembl Biomart (Ensembl genes 65) [98].

### Quantitative Reverse Transcriptase PCR (qRT-PCR)

Quantitative real time reverse transcriptase PCR (qRT-PCR) was performed on the same biological samples as those used for microarrays. Twenty-one target genes were selected to represent a wide range of microarray expressions from highest to lowest ratio from 17 to 1.2. Additionally, expression of ovocleidin-116 was also measured on the same samples. Total RNA samples (10 μg) used for microarrays experiments, were subjected to reverse transcription (RT) using MMLV RNase H– reverse transcriptase (Superscript II, Invitrogen, Illkirch, France) and random hexamers (Promega, Charbonnières les Bains, France). A 1:200 dilution of the RT product was use for real time amplification using LightCycler 480 SYBR Green I Master (Roche Applied Science, Mannheim, Germany) as recommended by the manufacturer’s instructions, and in presence of sense and anti-sense specific primers specific (Additional file 5), designed with Primer3Plus [99] to amplify 100 to 250 base pair (bp) amplicons. PCR conditions using the LightCycler 480 (Roche Applied Science, Mannheim, Germany) were as follows: A thermal denaturation step of the polymerase (95°C/10 min) followed by 45 cycles of amplification (denaturation: 95°C/10 sec, annealing: 60°C/20 sec, and elongation: 60°C/10 sec) with measurement of the emitted fluorescence at the end of each cycle. A melting curve (60°C to 95°C) was also performed to verify the presence of a single product with a specific melting temperature. Each run of PCR consisted of triplicates uterine samples, and contained “no template” controls without cDNA.

A standard curve was determined using a pool of 16 uterine RT products at serial dilutions. Calculation of mRNA levels was based on the detection of threshold cycle and the PCR efficiency derived from the standard curve. PCR amplification products were verified by electrophoresis and sequencing. To account for variations in RNA extraction and RT reaction, RNA levels were corrected by amplification of a reference gene, the TATA box binding protein (TBP), on the same samples. The ratio value was calculated for each sample as sample/TBP.

Statistical analysis was performed using a two-tailed Welch’s t-test to determine differences (p < 5%) between N and E uterine gene expression.

### Functional analysis

Biological interpretations were carried out using the Gene Ontology (GO) public database [47]. Enrichment of terms was carried out using Genomatix Pathway System (GePS) from the Genomatix suite [32] using a statistical threshold of 5%.

The protein sequences depicted from the differentially expressed transcripts were analyzed for the presence of a signal peptide (classical secretion pathway) using the SignalP 4.0 program [100]. Transmembrane proteins were predicted using TMHMM 2.0 program [101]. YLoc, an alternative prediction system for protein subcellular localization, was also performed to predict potentially extracellular proteins. This method combines *ab initio* predictors and functional annotations originating from PROSITE [102] and GO databases. Proteins involved in ionic transport were identified using GO « ion transport » terms and protein annotations in the Chicken Gene Nomenclature [48]. Proteomic eggshell protein sequences [14-18], reported as 568 different IPI identifiers (International Protein Index) which is no longer available, were searched to determine their actual corresponding sequences in protein databases (NCBI nr, Ensembl protein) for Gallus gallus species (Taxid: 9031). The following software were used: NCBI protein blast [97] and the Ensembl Biomart (Ensembl genes 65) [98]. Protein sequences were then linked to the gene products identified in the current transcriptomic analysis.

### Availability of supporting data

The microarray data were deposited in the NCBI Gene Expression Omnibus (GEO) data repository under accession number GSE 52823 [103].

## Abbreviations

BF: Bonferroni procedure; BH: Benjamini-Hochberg procedure; E: Expelled condition; N: Normal condition; GO: Gene Ontology; qRT-PCR: Quantitative real time RT-PCR.

## Competing interests

The authors declare that they have no competing interests.

## Authors’ contributions

AB was involved in designing and planning of the study. He carried out the experiments and analyses, interpreted data, annotation and statistical analyses and wrote the first draft of the paper. CHA was involved in the experimental design, performed the statistical analysis, and contributed to the writing of the paper. YN conceived the research program focused on identification of egg proteins. He was involved in the experimental design, data interpretation and in the writing of the paper. JG conceived the strategy, designed and experiments, interpreted data, annotation and statistical analyses and was fully involved in the writing of the paper. All authors have read and approved the final manuscript.

## Supplementary Material

Additional file 1 **Differentially expressed genes in hen uterus with calcifying shell.** Excel file describing the annotated and non-annotated differentially expressed genes. Results were expressed as log 2 N/E ratio. Click here for file

Additional file 2 **Comparison of gene expression from microarray and qRT-PCR analyses.** Excel file showing the level of expression and the statistical significances of selected genes for validation using qRT-PCR, as follows: ≤ 0.1 (†) 0.05 (*), 0.01 (**) 0.001 (***), not significant (ns). Click here for file

Additional file 3 **GO terms significantly enriched in the uterus transcriptome.** Excel file reporting GO terms (biological process and molecular function), significantly enriched and classified in various groups according to their functions. Click here for file

Additional file 4 **Comparison of the proteins derived from transcriptome analysis with those identified by proteomic studies of eggshell.** Excel file presenting the already identified eggshell proteins not present on the microarray, present on microarray but not expressed, present on microarray and differentially expressed during shell calcification, and proteins present on microarray but not differentially expressed. Click here for file

Additional file 5 **List of primers used for quantitative real-time RT-PCR.** Exel file indicating the gene identifiers and the sense and antisense primers used in this study. Click here for file

## References

[B1] Mine Y Kovacks-Nolan J New insights in biologically active proteins and peptides derived from hen egg World’s Poultry Sci J 2006 15 87 95 10.1079/WPS200586

[B2] Réhault-Godbert S Hervé V Gautron J Cabau C Nys Y Nys Y, Bain M, van Immerseel F Molecules involved in chemical defence of the chicken egg Improving the safety and quality of eggs and egg products Egg chemistry, production and consumption, Volume 1 2011 Cambridge: Woodhead Publishing limited 183 208

[B3] Mine Y D’Silva I Mine Y Bioactive components in egg white Egg bioscience and biotechnology 2008 Hoboken, New Jersey: John Wiley & Sons 141 184

[B4] Gautron J Nys Y Huopalahti R, Lopez-Fandino R, Anton M, Schade R Function of eggshell matrix proteins Bioactive egg compounds 2007 Germany: Springer-Verlag 109 115

[B5] Gautron J Nys Y Huopalahti R, Lopez-Fandino R, Anton M, Schade R Eggshell matrix proteins Bioactive egg compounds 2007 Germany: Springer-Verlag 103 108

[B6] Hincke MT Nys Y Gautron J The role of matrix proteins in eggshell formation J Poul Sci 2010 15 3 208 219 10.2141/jpsa.009122

[B7] Hincke MT Nys Y Gautron J Mann K Rodriguez-Navarro AB McKee MD The eggshell: structure, composition and mineralization Front Biosci 2012 15 1266 1280 10.2741/3985 22201802

[B8] Nys Y Gautron J Garcia-Ruiz JM Hincke MT Avian eggshell mineralization: biochemical and functional characterization of matrix proteins C R Palevol 2004 15 6–7 549 562

[B9] Tullet SG Wells RG, Belyavin CG Egg shell formation and quality Egg quality current problems and recent advances 1987 London: Butterworth 123 146

[B10] Gautron J Hincke MT Nys Y Precursor matrix proteins in the uterine fluid change with stages of eggshell formation in hens Connect Tissue Res 1997 15 3 195 210 10.3109/03008209709160220 9512888

[B11] Nys Y Guyot N Nys Y, Bain M, van Immerseel F Egg formation and chemistry Improving the Safety and Quality of Eggs and egg Products Egg chemistry, production and consumption, Volume 1 2011 Cambridge: Woodhead Publishing limited 83 132

[B12] Weiner S Biomineralization: a structural perspective J Struct Biol 2008 15 3 229 234 10.1016/j.jsb.2008.02.001 18359639

[B13] Weiner S Addadi L Clarke DR, Fratzl P Crystallization pathways in biomineralization Annual Review of Materials Research, Vol 41 2011 Annual Reviews, 4139 El Camino Way, Po Box 10139, Palo Alto, Ca 94303-0897 USA 21 40

[B14] Mann K Macek B Olsen JV Proteomic analysis of the acid-soluble organic matrix of the chicken calcified eggshell layer Proteomics 2006 15 13 3801 3810 10.1002/pmic.200600120 16767793

[B15] Mann K Olsen JV Macek B Gnad F Mann M Phosphoproteins of the chicken eggshell calcified layer Proteomics 2007 15 1 106 115 10.1002/pmic.200600635 17152097

[B16] Miksik I Eckhardt A Sedlakova P Mikulikova K Proteins of insoluble matrix of Avian (Gallus Gallus) eggshell Connect Tissue Res 2007 15 1 1 8 10.1080/03008200601003116 17364661

[B17] Miksik I Sedlakova P Lacinova K Pataridis S Eckhardt A Determination of insoluble avian eggshell matrix proteins Anal Bioanal Chem 2009 15 1 205 214 19998026 10.1007/s00216-009-3326-3

[B18] Rose-Martel M Jingwen D Hincke MT Proteomic analysis provides new insight into the chicken eggshell cuticle J Proteomics 2012 15 9 2697 2706 10.1016/j.jprot.2012.03.019 22708129

[B19] Jonchere V Rehault-Godbert S Hennequet-Antier C Cabau C Sibut V Cogburn LA Nys Y Gautron J Gene expression profiling to identify eggshell proteins involved in physical defense of the chicken egg BMC Genomics 2010 15 57 10.1186/1471-2164-11-57 PMC2827412 20092629

[B20] Nys Y Zawadzki J Gautron J Mills AD Whitening of brown-shelled eggs - mineral-composition of uterine fluid and rate of protoporphyrin deposition Poult Sci 1991 15 5 1236 1245 10.3382/ps.0701236 1852696

[B21] Eastin WC Jr Spaziani E On the mechanism of calcium secretion in the avian shell gland (uterus) Biol Reprod 1978 15 3 505 518 10.1095/biolreprod19.3.505 719101

[B22] Nys Y Hincke MT Arias JL Garcia-Ruiz JM Solomon SE Avian eggshell mineralization Avian Poult Biol Rev 1999 15 3 143 166

[B23] Jonchere V Brionne A Gautron J Nys Y Identification of uterine ion transporters for mineralisation precursors of the avian eggshell BMC Physiol 2012 15 10 10.1186/1472-6793-12-10 22943410 PMC3582589

[B24] Nys Y Sharp PJ Regulation of 1,25 (OH)2D3, of osteocalcin and of intestinal and uterine calbindin in hens Avian Endocrinology 1993 Bristol (UK): Journal of Endocrinology 345 357

[B25] Nys Y Nguyen TM Williams J Etches RJ Blood-levels of ionized calcium, inorganic phosphorus, 1,25-dihydroxycholecalciferol and gonadal-hormones in hens laying hard-shelled or shell-less eggs J Endocrinol 1986 15 1 151 157 10.1677/joe.0.1110151 3783081

[B26] Benjamini Y Hochberg Y Controlling the false discovery rate - a practical and powerful approach to multiple testing J R Statist Soc B 1995 15 1 289 300

[B27] Hillier LW Miller W Birney E Warren W Hardison RC Ponting CP Bork P Burt DW Groenen MAM Delany ME Dodgson JB Chinwalla AT Cliften PF Clifton SW Delehaunty KD Fronick C Fulton RS Graves TA Kremitzki C Layman D Magrini V McPherson JD Miner TL Minx P Nash WE Nhan MN Nelson JO Oddy LG Pohl CS Randall-Maher J Sequence and comparative analysis of the chicken genome provide unique perspectives on vertebrate evolution Nature 2004 15 7018 695 716 10.1038/nature03154 15592404

[B28] Ensembl Gallus gallus [http://www.ensembl.org/Gallus_gallus/Info/Index]

[B29] Benson DA Cavanaugh M Clark K Karsch-Mizrachi I Lipman DJ Ostell J Sayers EW GenBank Nucleic Acids Res 2013 15 D1 D36 D42 10.1093/nar/gks1195 27899564 PMC5210553

[B30] Entrez gene statistics [http://www.ncbi.nlm.nih.gov/projects/Gene/gentrez_stats.cgi?TAXORG=9031]

[B31] Entrez gene [http://www.ncbi.nlm.nih.gov/gene/]

[B32] Genomatix [http://www.genomatix.de]

[B33] International protein index [http://www.ebi.ac.uk/IPI]

[B34] Flicek P Ahmed I Amode MR Barrell D Beal K Brent S Carvalho-Silva D Clapham P Coates G Fairley S Fitzgerald S Gil L García-Girón C Gordon L Hourlier T Hunt S Juettemann T Kähäri AK Keenan S Komorowska M Kulesha E Longden I Maurel T McLaren WM Muffato M Nag R Overduin B Pignatelli M Pritchard B Pritchard E Ensembl 2013 Nucleic Acids Res 2013 15 D1 D48 D55 10.1093/nar/gks1236 23203987 PMC3531136

[B35] Uniprot [http://www.uniprot.org/]

[B36] SignalP 4.1 server [http://www.cbs.dtu.dk/services/SignalP/]

[B37] TMHMM server 2.0 [http://www.cbs.dtu.dk/services/TMHMM/]

[B38] YLoc [http://abi.inf.uni-tuebingen.de/Services/YLoc/webloc.cgi]

[B39] Briesemeister S Rahnenfuhrer J Kohlbacher O Going from where to why-interpretable prediction of protein subcellular localization Bioinformatics 2010 15 9 1232 1238 10.1093/bioinformatics/btq115 20299325 PMC2859129

[B40] Briesemeister S Rahnenfuhrer J Kohlbacher O YLoc-an interpretable web server for predicting subcellular localization Nucleic Acids Res 2010 15 W497 W502 10.1093/nar/gkq477 20507917 PMC2896088

[B41] Addadi L Weiner S Control and design principles in biological mineralization Angew Chem Int Ed 1992 15 153 169 10.1002/anie.199201531

[B42] Bar A Striem S Mayelafshar S Lawson DEM Differential regulation of calbindin-d28k messenger-rna in the intestine and eggshell gland of the laying hen J Mol Endocrinol 1990 15 2 93 99 10.1677/jme.0.0040093 2344392

[B43] Nys Y Mayelafshar S Bouillon R Vanbaelen H Lawson DEM Increases in calbindin d-28 k messenger-rna in the uterus of the domestic-fowl induced by sexual maturity and shell formation Gen Comp Endocrinol 1989 15 2 322 329 10.1016/0016-6480(89)90164-0 2591722

[B44] Jeong W Lim W Kim J Ahn SE Lee HC Jeong JW Han JY Song G Bazer FW Cell-specific and temporal aspects of gene expression in the chicken oviduct at different stages of the laying cycle Biol Reprod 2012 15 6 172 10.1095/biolreprod.111.098186 22423054

[B45] Lavelin I Meiri N Einat M Genina O Pines M Mechanical strain regulation of the chicken glypican-4 gene expression in the avian eggshell gland Am J Physiol Regul Integr Comp Physiol 2002 15 4 R853 R861 12228054 10.1152/ajpregu.00088.2002

[B46] Lavelin I Yarden N Ben-Bassat S Bar A Pines M Regulation of osteopontin gene expression during Egg shell formation in the laying Hen by mechanical strain Matrix Biol 1998 15 8–9 615 623 9923654 10.1016/s0945-053x(98)90112-3

[B47] Ashburner M Ball CA Blake JA Botstein D Butler H Cherry JM Davis AP Dolinski K Dwight SS Eppig JT Harris MA Hill DP Issel-Tarver L Kasarskis A Lewis S Matese JC Richardson JE Ringwald M Rubin GM Sherlock G Gene Ontology: tool for the unification of biology Nat Genet 2000 15 1 25 29 10.1038/75556 10802651 PMC3037419

[B48] Burt DW Carre W Fell M Law AS Antin PB Maglott DR Weber JA Schmidt CJ Burgess SC McCarthy FM The chicken gene nomenclature committee report BMC Genomics 2009 15 S5 19607656 10.1186/1471-2164-10-S2-S5 PMC2966335

[B49] Bar A Calcium transport in strongly calcifying laying birds: mechanisms and regulation Comp Biochem Physiol A Mol Integr Physiol 2009 15 4 447 469 10.1016/j.cbpa.2008.11.020 19118637

[B50] Tempel BL Shilling DJ The plasma membrane calcium ATPase and disease Subcell Biochem 2007 15 365 383 10.1007/978-1-4020-6191-2_13 18193644

[B51] Lorcher K Hodges RD Some possible mechanisms of formation of the carbonate fraction of egg shell calcium carbonate Comp Biochem Physiol 1969 15 1 119 128 10.1016/0010-406X(69)91326-7 5777361

[B52] Gay CV Mueller WJ Cellular localization of carbonic anhydrase in avian tissues by labeled inhibitor autoradiography J Histochem Cytochem 1973 15 8 693 702 10.1177/21.8.693 4199175

[B53] Gay CV Faleski EJ Schraer H Schraer R Localization of carbonic anhydrase in avian gastric mucosa, shell gland and bone by immunohistochemistry J Histochem Cytochem 1974 15 8 819 825 10.1177/22.8.819 4212144

[B54] Gilmour KM Perspectives on carbonic anhydrase Comp Biochem Physiol A Mol Integr Physiol 2010 15 3 193 197 10.1016/j.cbpa.2010.06.161 20541618

[B55] Lavelin I Meiri N Genina O Alexiev R Pines M Na + −K + −ATPase gene expression in the avian eggshell gland: distinct regulation in different cell types Am J Physiol Regul Integr Comp Physiol 2001 15 4 R1169 R1176 11557625 10.1152/ajpregu.2001.281.4.R1169

[B56] Gorokhova S Bibert S Geering K Heintz N A novel family of transmembrane proteins interacting with beta subunits of the Na, K-ATPase Hum Mol Genet 2007 15 2394 2410 10.1093/hmg/ddm167 17606467

[B57] Gao L Cong BG Zhang LM Ni X Expression of the calcium-activated potassium channel in upper and lower segment human myometrium during pregnancy and parturition Reprod Biol Endocrinol 2009 15 27 10.1186/1477-7827-7-27 19344525 PMC2670306

[B58] Greenwood IA Yeung SY Tribe RM Ohya S Loss of functional K + channels encoded by ether-a-go-go-related genes in mouse myometrium prior to labour onset J Physiol 2009 15 10 2313 2326 10.1113/jphysiol.2009.171272 19332483 PMC2697300

[B59] Beyenbach KW Wieczorek H The V-type H + ATPase: molecular structure and function, physiological roles and regulation J Exp Biol 2006 15 4 577 589 10.1242/jeb.02014 16449553

[B60] Ding ST Chang CC Shen TF The effect of dietary magnesium and calcium level on the eggshell quality and mineral-content in plasma, eggshell and bone in laying Ttsaiya duck and leghorn hen J Agric Assoc China 1992 15 97 107

[B61] Goytain A Quamme GA Functional characterization of the human solute carrier, SLC41A2 Biochem Biophys Res Commun 2005 15 3 701 705 10.1016/j.bbrc.2005.03.037 15809054

[B62] Sahni J Nelson B Scharenberg AM SLC41A2 encodes a plasma-membrane Mg2+ transporter Biochem J 2007 15 505 513 10.1042/BJ20060673 16984228 PMC1820800

[B63] Moomaw AS Maguire ME The Unique Nature of Mg2+ Channels Physiology 2008 15 5 275 285 10.1152/physiol.00019.2008 18927203 PMC2711038

[B64] Ebel H Gunther T Magnesium metabolism - a review J Clin Chem Clin Biochem 1980 15 5 257 270 7000968 10.1515/cclm.1980.18.5.257

[B65] Goytain A Hines RM Quamme GA Functional characterization of NIPA2, a selective Mg2+ transporter Am J Physiol Cell Physiol 2008 15 4 C944 C953 10.1152/ajpcell.00091.2008 18667602

[B66] Turski ML Thiele DJ New roles for copper metabolism in cell proliferation, signaling, and disease J Biol Chem 2009 15 2 717 721 10.1074/jbc.R800055200 18757361 PMC2613604

[B67] Knutson MD Steap proteins: implications for iron and copper metabolism Nutr Rev 2007 15 7 335 340 10.1301/nr.2007.jul.335–340 17695374

[B68] Ohgami RS Campagna DR McDonald A Fleming MD The Steap proteins are metalloreductases Blood 2006 15 4 1388 1394 10.1182/blood-2006-02-003681 16609065 PMC1785011

[B69] Haguenauer A Raimbault S Masscheleyn S Gonzalez-Barroso MD Criscuolo F Plamondon J Miroux B Ricquier D Richard D Bouillaud F Pecqueur C A new renal mitochondrial carrier, KMCP1, is up-regulated during tubular cell regeneration and induction of antioxidant enzymes J Biol Chem 2005 15 23 22036 22043 10.1074/jbc.M412136200 15809292

[B70] Palmieri F The mitochondrial transporter family (SLC25): physiological and pathological implications Pflugers Arch 2004 15 5 689 709 10.1007/s00424-003-1099-7 14598172

[B71] Mann K The chicken egg white proteome Proteomics 2007 15 3558 3568 10.1002/pmic.200700397 17722208

[B72] Nickel W Pathways of unconventional protein secretion Curr Opin Biotechnol 2010 15 5 621 626 10.1016/j.copbio.2010.06.004 20637599

[B73] Hincke MT Chien Y-C Gerstenfeld LC McKee MD Colloidal-gold immunocytochemical localization of osteopontin in avian eggshell gland and eggshell J Histochem Cytochem 2008 15 5 467 476 10.1369/jhc.2008.950576 18256019 PMC2324194

[B74] Pines M Knopov V Bar A Involvement of osteopontin in egg shell formation in the laying chicken Matrix Biol 1995 15 9 765 771 10.1016/S0945-053X(05)80019-8 8785591

[B75] Chien YC Hincke MT Vali H McKee MD Ultrastructural matrix-mineral relationships in avian eggshell, and effects of osteopontin on calcite growth in vitro J Struct Biol 2008 15 1 84 99 10.1016/j.jsb.2008.04.008 18511297

[B76] Hincke MT St Maurice M Goldberg M, Boskey A, Robinson C phosphorylation-dependent modulation of calcium carbonate precipitation by chicken eggshell matrix proteins Chemistry and Biology of Mineralized Tissues 2000 Rosemont, IL 60018: American Academy of Orthopaedic Surgeons 13 17

[B77] Hincke MT Gautron J Tsang CP McKee MD Nys Y Molecular cloning and ultrastructural localization of the core protein of an eggshell matrix proteoglycan, ovocleidin-116 J Biol Chem 1999 15 46 32915 32923 10.1074/jbc.274.46.32915 10551857

[B78] Mann K Hincke MT Nys Y Isolation of ovocleidin-116 from chicken eggshells, correction of its amino acid sequence and identification of disulfide bonds and glycosylated Asn Matrix Biol 2002 15 5 383 387 10.1016/S0945-053X(02)00031-8 12225802

[B79] Bardet C Delgado S Sire JY MEPE evolution in mammals reveals regions and residues of prime functional importance Cell Mol Life Sci 2010 15 2 305 320 10.1007/s00018-009-0185-1 19924383 PMC11115541

[B80] Rosen H Polakiewicz RD Benzakine S Barshavit Z Proenkephalin-A in bone-derived cells Proc Natl Acad Sci U S A 1991 15 9 3705 3709 10.1073/pnas.88.9.3705 2023920 PMC51521

[B81] Keene DR San Antonio JD Mayne R McQuillan DJ Sarris G Santoro S Iozzo RV Decorin binds near the C terminus of type I collagen J Biol Chem 2000 15 29 21801 21804 10.1074/jbc.C000278200 10823816

[B82] Sullivan MM Barker TH Funk SE Karchin A Seo NS Hook M Sanders J Starcher B Wight TN Puolakkainen P Sage EH Matricellular hevin regulates decorin production and collagen assembly J Biol Chem 2006 15 37 27621 27632 10.1074/jbc.M510507200 16844696

[B83] Fernandez MS Araya M Arias JL Eggshells are shaped by a precise spatio-temporal arrangement of sequentially deposited macromolecules Matrix Biol 1997 15 1 13 20 10.1016/S0945-053X(97)90112-8 9181550

[B84] Fernandez MS Moya A Lopez L Arias JL Secretion pattern, ultrastructural localization and function of extracellular matrix molecules involved in eggshell formation Matrix Biol 2001 15 8 793 803 10.1016/S0945-053X(00)00128-1 11223339

[B85] Ito A Shinmyo Y Abe T Oshima N Tanaka H Ohta K Tsukushi is required for anterior commissure formation in mouse brain Biochem Biophys Res Commun 2010 15 4 813 818 10.1016/j.bbrc.2010.10.127 21055390

[B86] Filmus J Capurro M Rast J Glypicans Genome Biol 2008 15 5 224 10.1186/gb-2008-9-5-224 18505598 PMC2441458

[B87] Apte SS A disintegrin-like and metalloprotease (reprolysin-type) with thrombospondin type 1 motif (ADAMTS) superfamily: functions and mechanisms J Biol Chem 2009 15 46 31493 31497 10.1074/jbc.R109.052340 19734141 PMC2797218

[B88] Mann K Gautron J Nys Y McKee MD Bajari T Schneider WJ Hincke MT Disulfide-linked heterodimeric clusterin is a component of the chicken eggshell matrix and egg white Matrix Biol 2003 15 5 397 407 10.1016/S0945-053X(03)00072-6 14614987

[B89] Gautron J Murayama E Vignal A Morisson M McKee MD Rehault S Labas V Belghazi M Vidal ML Nys Y Hincke MT Cloning of ovocalyxin-36, a novel chicken eggshell protein related to lipopolysaccharide-binding proteins, bactericidal permeability-increasing proteins, and plunc family proteins J Biol Chem 2007 15 8 5273 5286 10.1074/jbc.M610294200 17179153

[B90] Bingle CD Seal RL Craven CJ Systematic nomenclature for the PLUNC/PSP/BSP30/SMGB proteins as a subfamily of the BPI fold-containing superfamily Biochem Soc Trans 2011 15 977 983 10.1042/BST0390977 21787333 PMC3196848

[B91] Harada H Ohto U Satow Y Crystal structure of mouse MD-1 with endogenous phospholipid bound in its cavity J Mol Biol 2010 15 4 838 846 10.1016/j.jmb.2010.05.063 20595044

[B92] Rehault-Godbert S Labas V Helloin E Herve-Grepinet V Slugocki C Berges M Bourin M-C Brionne A Poirier J-C Gautron J Coste F Nys Y Ovalbumin-related protein X is a heparin-binding Ov-Serpin exhibiting antimicrobial activities J Biol Chem 2013 15 24 17285 17295 10.1074/jbc.M113.469759 23615912 PMC3682532

[B93] Réhault-Godbert S Jousset N Labas V Hervé-Grépinet V Nys Y Gautron J Identification of putative functional proteins in chicken eggshell XXIII World’s Poultry Congress: 30 June - 07 July 2008 2008 2008 Brisbane (AUS; World\'s Poultry Science Association (Australian branch) 114 118

[B94] CRB GADIE [http://crb-gadie.inra.fr]

[B95] Limma R package [http://www.bioconductor.org/packages/2.12/bioc/html/limma.html]

[B96] Smyth GK Linear models and empirical bayes methods for assessing differential expression in microarray experiments Stat Appl Genet Mol Biol 2004 15 Article3 16646809 10.2202/1544-6115.1027

[B97] Sayers EW Barrett T Benson DA Bolton E Bryant SH Canese K Chetvernin V Church DM DiCuccio M Federhen S Feolo M Geer LY Helmberg W Kapustin Y Landsman D Lipman DJ Lu ZY Madden TL Madej T Maglott DR Marchler-Bauer A Miller V Mizrachi I Ostell J Panchenko A Pruitt KD Schuler GD Sequeira E Sherry ST Shumway M Database resources of the National Center for Biotechnology Information Nucleic Acids Res 2011 15 D5 D16 10.1093/nar/gkp967 PMC2808881 19910364

[B98] Kinsella RJ Kahari A Haider S Zamora J Proctor G Spudich G Almeida-King J Staines D Derwent P Kerhornou A Kersey P Flicek P Ensembl BioMarts: a hub for data retrieval across taxonomic space Database 2011 15 bar030 21785142 10.1093/database/bar030 PMC3170168

[B99] Primer3Plus software [http://www.bioinformatics.nl/cgi-bin/primer3plus/primer3plus.cgi]

[B100] Petersen TN Brunak S von Heijne G Nielsen H SignalP 4.0: discriminating signal peptides from transmembrane regions Nat Meth 2011 15 10 785 786 10.1038/nmeth.1701 21959131

[B101] Emanuelsson O Brunak S von Heijne G Nielsen H Locating proteins in the cell using TargetP, SignalP and related tools Nat Protoc 2007 15 4 953 971 10.1038/nprot.2007.131 17446895

[B102] Sigrist CJA Cerutti L de Castro E Langendijk-Genevaux PS Bulliard V Bairoch A Hulo N PROSITE, a protein domain database for functional characterization and annotation Nucleic Acids Res 2010 15 D161 D166 10.1093/nar/gkp885 19858104 PMC2808866

[B103] Gene expression omnibus [http://www.ncbi.nlm.nih.gov/geo]

